# Improving glass Ionomer performance through plant extracts: a systematic review of *in vitro* studies

**DOI:** 10.2340/biid.v12.45152

**Published:** 2025-12-29

**Authors:** Israel Luís Diniz Carvalho, Maria Helena Nunes Borges, Geisa Aiane de Morais Sampaio, Gabriela Queiroz de Melo Monteiro, Moan Jéfter Fernandes Costa, Bruno Lima, José Roberto de Oliveira Bauer, Pedro Henrique Sette-de-Souza

**Affiliations:** a Graduate Program in Dentistry, University of Pernambuco – Campus Santo Amaro, Recife, PE, Brazil; b Multiuser Laboratory of Biomaterials from the Caatinga (BIOMA), University of Pernambuco, Arcoverde, PE, Brazil; c Department of Endodontics, University of Florida, Gainesville, FL, USA; d Graduate Program in Dentistry, Federal University of Maranhão, São Luís, MA, Brazil

**Keywords:** Resin-modified GIC, cytotoxicity, physical properties, glass ionomer cement, antibacterial properties, physicochemical properties, plant extract

## Abstract

**Objective:**

This systematic review aimed to systematically map and synthesize the available evidence from *in vitro* studies on the modification of Glass Ionomer Cements (GICs) with plant extracts, with a specific focus on evaluating their effects on the material’s antibacterial, physicochemical, and mechanical properties.

**Materials and methods:**

The review was conducted in accordance with the Preferred Reporting Items for Systematic reviews and Meta-Analyses extension for Scoping Reviews (PRISMA-ScR) guidelines. The search strategy, based on the Population Intervention, Comparison, and Outcome (PICO) framework, was applied to four major databases (PubMed, Embase, Scopus, and Web of Science), yielding 2,614 initial records. The Quality Assessment Tool for In Vitro Studies (QUINN Tool) was used to evaluate the risk of bias. Data regarding plant species, GIC types, modification methods, and outcome measures were extracted and synthesized narratively due to methodological heterogeneity.

**Results:**

The analysis of the 14 included studies revealed that *Salvadora persica* was the most frequently used plant species (6 studies). The primary outcome was a significant enhancement of the antibacterial activity of GICs against *Streptococcus mutans* without negatively affecting fluoride release. Most studies reported maintained or improved mechanical properties, such as compressive strength, at low extract concentrations (typically below 5%). However, the review identified significant limitations: a lack of methodological detail in extract incorporation, a near absence of cytotoxicity assessments, and insufficient investigation into ion release profiles beyond fluoride.

**Conclusions:**

The incorporation of plant extracts presents a promising strategy for improving the antibacterial performance of GICs while preserving their beneficial properties. However, the current body of evidence is constrained by methodological inconsistencies and critical gaps in safety and long-term efficacy evaluation. Future research must prioritize standardized protocols, comprehensive biocompatibility testing, and analyses under conditions that better simulate the oral environment to ensure clinical translatability.

Key messages

Plant extract-modified GICs show significant inhibition of *Streptococcus mutans*, outperforming chlorhexidine in some studies.

Low concentrations (≤ 5%) of extracts maintain fluoride release and key physical properties like compressive strength.

Critical gaps remain in cytotoxicity assessment and long-term clinical performance, urging standardized future research.

## Introduction

Glass Ionomer Cement (GIC), developed by Wilson and Kent (1971), is a translucent restorative material composed of a calcium fluoride-containing aluminosilicate glass powder and polyacrylic acid (Saridena et al., 2022; Wilson & Kent, 1971). Since its development, GIC has undergone continuous improvements and has found extensive clinical application in restorations, sealants, liners, Atraumatic Restorative Treatment (ART), oral rehabilitation, and orthodontics (Makanjuola & Deb, 2023; Saridena et al., 2022). Its use is particularly recognized among vulnerable populations, who often exhibit high caries rates, thereby supporting public health systems, especially in developing countries, in addressing one of the most prevalent oral health problems: dental caries (Conti et al., 2023; Makanjuola & Deb, 2023).

GIC is widely employed in dentistry due to its favourable properties, including adhesion to dental tissues, release of fluoride and calcium ions which promote remineralization and caries prevention, as well as its biocompatibility and low toxicity (Ersahan et al., 2020; Makanjuola & Deb, 2023; Nicholson et al., 2023; Song et al., 2025). Its non-cytotoxicity is attributed to the release of non-harmful ions, minimal exothermic reaction, and short setting neutralization time (Ersahan et al., 2020). However, conventional GICs are limited by low mechanical strength, reduced longevity, susceptibility to fracture and salivary degradation, and surface porosity that favours microbial adhesion and compromises durability (Fierascu, 2022; Wulandari et al., 2022).

To address these shortcomings without compromising the beneficial properties of GICs, several materials have been explored as additives. The incorporation of chlorhexidine digluconate (CHX) aims to enhance antibacterial activity, although it may reduce fluoride release (Hassan et al., 2022). More recent approaches involving the functionalization of GIC particles with CHX have demonstrated improved performance without impairing ion release (Gomes et al., 2024). Nevertheless, CHX concentrations at or above 5% may lead to material degradation (Gomes et al., 2024). Inorganic nanoparticles, including silica (SiO₂), alumina (Al₂O₃), and titania (TiO₂), have shown significant improvements in mechanical properties such as compressive strength (CS) and microhardness (Güçlü et al., 2024; Meyer et al., 2025; Patil et al., 2025). However, these nanoparticles face challenges including particle agglomeration at higher concentrations, potential interference with the acid-base setting reaction, and possible cytotoxic effects (Güçlü et al., 2024; Meyer et al., 2025; Patil et al., 2025). Silver and copper nanoparticles have been shown to increase CS but are associated with cytotoxicity, discoloration, and time-dependent loss of antimicrobial effectiveness (Fierascu, 2022). Organic substances, such as fish scale powder, may enhance strength and reduce porosity at low concentrations; however, excessive amounts increase viscosity, impair handling, and may lead to cracking (Wulandari et al., 2022).

Medicinal plant extracts have emerged as promising candidates for enhancing the properties of GICs by contributing antimicrobial activity and representing safer and more affordable alternatives compared to other additives (Choukhachizadeh Linhares et al., 2022; Moghaddam et al., 2022; Singer et al., 2020a). This positions them as effective tools for caries prevention in underserved populations and as viable options for public healthcare systems (Choukhachizadeh Moghaddam et al., 2022; Linhares et al., 2022; Singer et al., 2020a). For instance, the incorporation of curcumin crystals has been shown to improve the antibacterial properties of GIC without increasing its solubility or water absorption (Choukhachizadeh Moghaddam et al., 2022). However, no additive has as yet succeeded in simultaneously optimizing all desirable properties of the material without adverse effects, underscoring the need for further studies involving novel plant species or new combinations of previously tested ones (Dutra et al., 2024).

Given the wide-ranging application of GICs in dentistry and the ongoing need to enhance their properties without undermining their clinical effectiveness, medicinal plant extracts represent a promising avenue. These extracts contain bioactive metabolites with antimicrobial effects and offer potential cost advantages compared to conventional additives, making them especially appealing for public healthcare services. Nevertheless, gaps remain in the literature concerning their integration with GICs. Therefore, the present study aims to consolidate existing knowledge, identify opportunities for innovation, and guide future research on the use of plant extracts to improve GICs while maintaining their safety and efficacy.

## Materials and methods

This systematic review was conducted in accordance with the PRISMA 2020 guidelines (Preferred Reporting Items for Systematic Reviews and Meta-Analyses) (Page et al., 2022). The review protocol was registered on the Open Science Framework – OSF (DOI: https://osf.io/3w7zn).

### Eligibility criteria

The following guiding question was adopted: ‘Do plant extracts improve the properties of Glass Ionomer Cements (GICs)?’ To structure the search, the PICO strategy was applied: GIC (Population), modified with plant extracts (Intervention), compared to conventional GICs and/or those modified with Chlorhexidine (Comparison), focusing on improvements in physicochemical and mechanical properties (Outcome).

Included studies were in vitro, peer-reviewed publications investigating the use of plant extracts as a strategy to enhance GIC properties. Only studies with a control group were considered eligible. Articles in any language and without publication year restriction were accepted. Exclusion criteria encompassed case reports, reviews, dissertations, monographs, letters to the editor, book chapters, and studies lacking a comparator group or not involving plant extracts.

### Information sources

The databases searched were: PubMed, Embase, Scopus, and Web of Science. Manual searches were also conducted using the reference lists of included studies, as well as the platforms Connected Papers and Google Scholar to identify additional potentially relevant records. No further studies were added through these supplementary sources.

### Search strategy

The search strategy was developed using standardized descriptors combined through Boolean operators, applied as follows:

(“Glass Ionomer Cement” OR “GIC” OR “dental materials” OR “modified GIC”) AND (“plant extracts” OR “herbal extracts” OR “phytochemicals”) AND (“mechanical properties” OR “antibacterial properties” OR “biocompatibility” OR “antibacterial test” OR “antimicrobial assay” OR “antimicrobial activity” OR “biofilm inhibition” OR “zone of inhibition” OR “compressive strength” OR “tensile strength” OR “flexural strength” OR “hardness test” OR “microhardness microleakage”).

No filters were applied for publication year or language. Each strategy was tailored to the syntax and operators of the specific database used.

### Study selection

Study selection was independently performed by two reviewers (P.H.S.S. and M.J.F.C.), and cross-checked by two additional reviewers (I.L.D.C. and M.H.N.B.) in two stages: screening of titles and abstracts, followed by full-text assessment of eligible articles. The Rayyan software was used to facilitate study screening and duplicate removal. Inter-reviewer agreement was assessed using the kappa coefficient (0.96). Discrepancies were resolved by consensus.

### Data collection process

Data extraction was also independently conducted by the same reviewers. The extracted information included: author names and nationalities, year of publication, journal impact factor, type of plant extract, bacterial strains analysed, GICs employed, extract incorporation method and ratio, specimen size, study limitations, key findings, and conclusions.

Antimicrobial outcomes and characterization were assessed using agar diffusion, Minimum Inhibitory Concentration (MIC), MTT viability assay, Antimicrobial Photodynamic Therapy (aPDT), and broth microdilution. Chemical characterization involved: GC-MS, UPLC-QTOF-MS/MS, spectrophotometry, UV-VIS, and Fourier-transform infrared spectroscopy (FTIR) with fluorometer. Physical characterization included: film thickness, Scanning Electron Microscopy (SEM), fracture mode, syneresis and imbibition, solubility, water sorption and solubility, surface microhardness, elastic modulus, CS, flexural strength, shear bond strength, Diametral Tensile Strength (DTS), and thermal analysis (TG/DTG/DTA).

### Risk of bias assessment

Risk of bias was assessed using the Quality Assessment Tool for In Vitro Studies (QUIN Tool) (Sheth et al., 2022). Each study was evaluated according to 12 methodological criteria: clearly defined objectives (Q1), sample size calculation (Q2), sampling technique (Q3), comparison group (Q4), detailed methodology (Q5), information about operators (Q6), randomization (Q7), outcome measurement method (Q8), outcome assessor details (Q9), blinding (Q10), statistical analysis (Q11), and result reporting (Q12).

Each criterion was rated as ‘adequately specified’ (2 points), ‘inadequately specified’ (1 point), ‘not specified’ (0 points), or ‘not applicable’. Final scores were calculated as: Final score = (Total score × 100) / (2 × 12).

Studies were classified as having low (> 70%), moderate (50–70%), or high (< 50%) risk of bias. Two reviewers (P.H.S.S. and I.L.D.C.) independently assessed each study; disagreements were resolved by consensus.

### Synthesis methods

The studies were qualitatively grouped based on the characteristics of population, intervention, comparators, and outcomes. Due to methodological heterogeneity, meta-analysis was not feasible. The results are presented in summary tables and descriptive narrative. Sensitivity analysis and statistical heterogeneity assessment were not applied.

## Results and discussion

### Study selection

**Figure 1:**
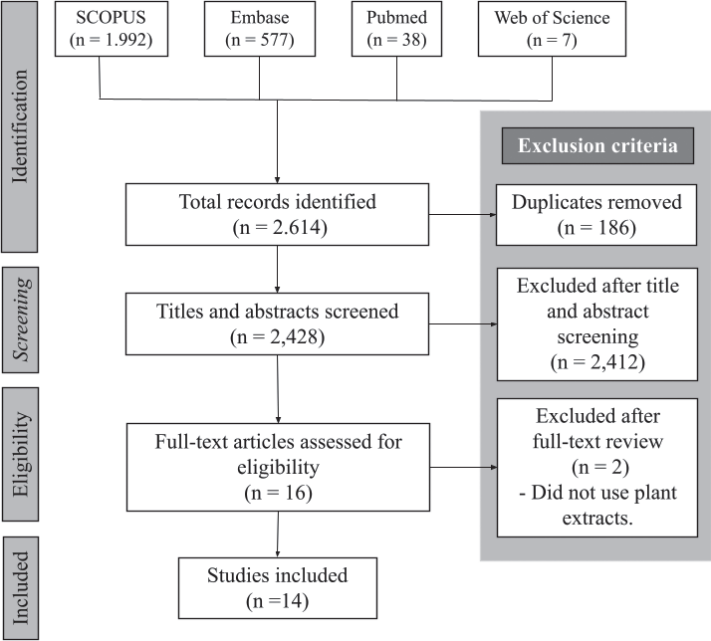
Flowchart detailing the steps of the search process..

Based on the search criteria described in the experimental section, a total of 2,614 studies were identified. After screening titles and abstracts, 16 articles were selected for full-text evaluation. Of these, two articles were excluded after a complete review for not meeting the inclusion criteria described in the experimental section, resulting in 14 studies included in the final analysis. The details of the search and selection process are illustrated in Flowchart 1 (Figure 1).

### Study characteristics

The 14 included studies were published in English between 2011 and 2024, with a concentration in the year 2020, accounting for six articles (42.9%) (Forouzanmehr & Barekatain; Mathew & Sghaireen, 2020; Paulraj & Nagar, 2020; Pinto et al., 2020; Shahtalebi, 2020; Singer et al., 2020a, 2020b). From 2019 onward, there was a noticeable increase in publications addressing the use of plant extracts to modify GIC (Table 1). Germany (Singer & Bourauel, 2021; Singer et al., 2020a, 2020b) and Brazil (Chiode et al., 2022; Pinto et al., 2020; Saraceni et al., 2019) stood out as leading contributors to this research field, each with three publications.

**Table 1:** Summary of included studies.

Authors	Year	Country	Plant	Family	Used GIC	Microbial Test	Microorganism	Physicochemical Characterization	Conclusion
El-Tatari et al.	2011	Netherlands	*Salvadora persica*	Salvadoraceae	GC Fuji IX	Agar diffusion test	*Candida albicans, Streptococcus mutans, Streptococcus sanguis, Streptococcus mitis, Streptococcus salivaruis and Actinomyces naeslundii*	Compressive strength and diametral tensile strength	Extract increased antimicrobial properties but concentrations > 1% reduced mechanical strength
Saraceni et al.	2019	Brazil	*Dioscorea altissima*	Dioscoreaceae	Vidrion R®	Broth microdilution + MTT	*S. mutans*	Syneresis/imbibition, solubility, elastic modulus, microhardness, fluoride release	Extract improved antimicrobial activity and microhardness
Shahriari et al.	2019	Iran	*Salvia officinalis*	Lamiaceae	GC Fuji IX	Agar diffusion test	*S. mutans; Lactobacillus casei*	-	Dose-dependent activity against S. mutans
Forouzanmehr et al.	2020	Iran	*S. officinalis*	Lamiaceae	Fuji II LC	-	*-*	Compressive strength and dentin bond strength	Did not alter mechanical properties
Mathew & Sghaireen	2020	Saudi Arabia	*Ziziphus spina-christi*	Rhamnaceae	Type II restorative GIC	Agar diffusion test	*S. mutans*	-	Inhibition only in direct contact
Paulraj & Nagar	2020	India	*Triphala (Phyllanthus emblica, Terminalia bellirica and Terminalia chebul)*	Phyllanthaceae and Combretaceae	Type IX GIC	Disk diffusion test	*S. mutans; Lactobacillus acidophilus*	-	Triphala significantly increased antimicrobial activity
Pinto et al.	2020	Brazil	*Schinus terebinthifolius*	Anacardiaceae	Maxxion C	Agar diffusion test	*S. aureus, S. mutans, Aggregatibacter actinomycetemcomitans and C. albicans*	UPLC, FTIR, Thermal analyses, SEM, compressive strength	Maintained antimicrobial activity with controlled release
Singer et al. (a)	2020	Germany	*Salvadora persica, Olea europaea and Ficus carica*	Salvadoraceae, Oleaceae and Moraceae	Medicem aqua	-	*-*	Water sorption/solubility, flexural strength	Improved flexural strength
Singer et al. (b)	2020	Germany	*S. persica, Olea europaea and Ficus carica*	Salvadoraceae, Oleaceae and Moraceae	Medicem aqua	Agar diffusion test	*S. mutans; Micrococcus luteus*	GC/MS and compressive strength	Improved antimicrobial activity and strength
Singer & Bourauel	2021	Germany	*S. persica, O. europaea and F. Carica*	Salvadoraceae, Oleaceae and Moraceae	Medicem aqua	-	*-*	Shear bond strength, film thickness	Did not compromise shear strength
Ashour et al.	2022	Saudi Arabia	*S. persica*	Salvadoraceae	GC Fuji IX	Agar diffusion test	*S. mutans; S. aureus and C. albicans*	FT-IR spectroscopy and compressive strength	Enhanced antimicrobial action without compromising mechanical properties
Chiode et al.	2022	Brazil	*D. altissima*	Dioscoreaceae	Vidrion R®	Bacterial viability test (MTT) + aPDT	*S. mutans*	UV-VIS spectrophotometry	Enhanced antimicrobial action and can replace methylene blue
Devi et al.	2022	India	*Swertia chirayita and Terminalia arjuna*	Gentianaceae and Combretaceae	GC Corporation IX	Minimum Inhibitory Concentration	*S. mutans and L. acidophilus*	Compressive strength	Improved antimicrobial activity without compromising strength
Siddiqui et al.	2024	Pakistan	*S. persica*	Salvadoraceae	GC Gold Label® Type II	Disk diffusion test	*S. mutans*	Vickers microhardness	3% ethanolic extract maximized activity and hardness

MTT: Methylthiazolyldiphenyl-tetrazolium bromide; UV-VIS: Ultraviolet-Visible Spectroscopy; aPDT: Antimicrobial Photodynamic Therapy; FT-IR: Fourier Transform Infrared Spectroscopy; GC/MS: Gas Chromatography-Mass Spectrometry; UPLC: Ultra Performance Liquid Chromatography; SEM: Scanning Electron Microscopy.

**Figure 2:**
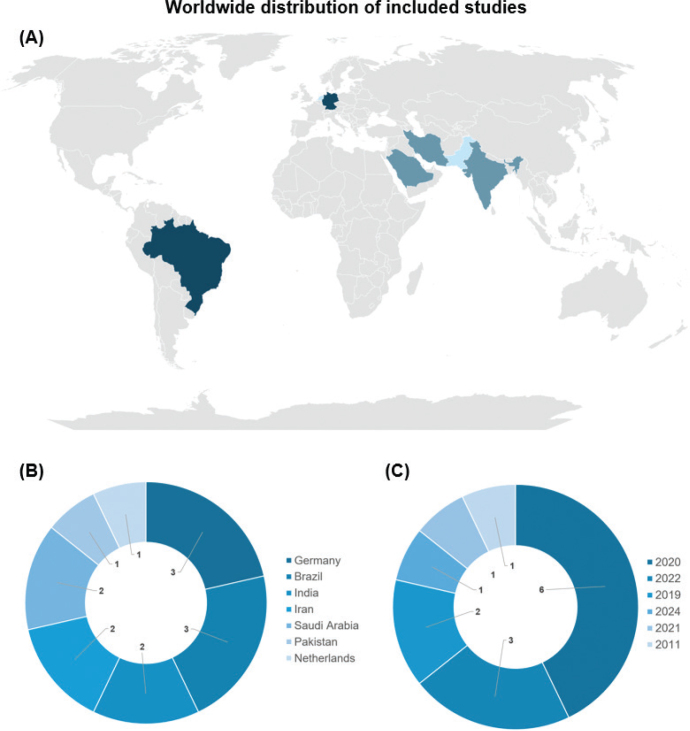
Geographic distribution of the selected studies. (A) Spatial distribution; (B) Number of included studies by the corresponding author’s country; (C) Year of publication.

The majority of studies – 10 out of 14 (71.4%) – were conducted in developing countries (Ashour et al., 2022; Barekatain & Shahtalebi, 2020; Chiode et al., 2022; Devi et al., 2022; Forouzanmehr & Mathew & Sghaireen, 2020; Paulraj & Nagar, 2020; Pinto et al., 2020; Saraceni et al., 2019; Shahriari et al., 2019; Siddiqui et al., 2024). Notably, no studies from the United States or China were identified in this review (Figure 2). This finding contrasts with broader trends in dental research, in which high-income countries such as the United States have traditionally led both in publication output and citation impact, and China has shown exponential growth in scientific production since 2020 (Daryakenari & Batooli, 2022; Mayta-Tovalino et al., 2023; Xie et al., 2024).

In the context of material modification strategies, it is noteworthy that the incorporation of chlorhexidine into GIC has been investigated for over two decades, since the works of Sanders et al. (2002) and Palmer et al. (2004). However, despite the extensive body of research on this compound, advances in the field have occurred at a relatively modest pace, and its clinical benefits remain limited. The incorporation of chlorhexidine has been associated with a short-term antimicrobial effect and, in some cases, with a potential negative impact on the mechanical stability and longevity of restorations (Mota-Martins et al., 2022). In contrast, the incorporation of complex plant extracts into GICs represents a more recent and promising approach, reflecting a broader scientific interest in natural bioactive agents capable of providing sustained antimicrobial activity with minimal interference in the material’s physicochemical integrity. As shown by the temporal analysis of publications presented in this study, research on plant-derived compounds in GICs has intensified mainly in the last few years, suggesting a paradigm shift toward more biologically driven strategies to enhance the performance of these restorative materials.

### Risk of bias in the studies

**Figure 3:**
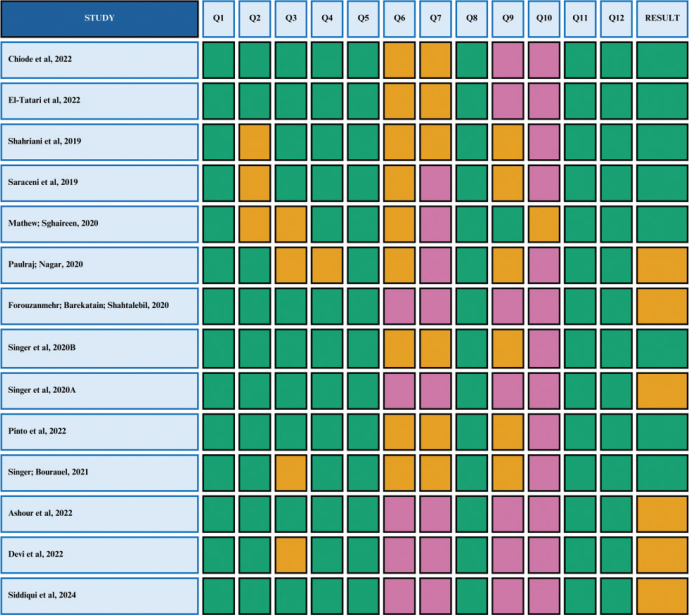
Risk of bias analysis (QUIN Tool). Q1: Clearly defined objectives; Q2: Sample size calculation; Q3: Sampling technique; Q4: Comparison group; Q5: Detailed methodological description; Q6: Operator details; Q7: Randomization; Q8: Outcome measurement method; Q9: Outcome assessor details; Q10: Blinding; Q11: Statistical analysis; Q12: Results presentation. Green: low risk; Orange: moderate risk; Purple: high risk.

The QUINN tool was used to assess the risk of bias. Most of the studies analyzed were classified as having low or moderate risk of bias (Figure 3). Of the 14 included studies, eight (57.1%) achieved a score above 70% and were categorized as low risk. The remaining studies were rated as having moderate risk. No study was classified as high risk of bias. Recurring methodological shortcomings were observed in the domains related to ‘details of outcome assessors’, ‘blinding’, and ‘randomization’, highlighting limitations in both the execution and reporting of these experimental elements.

Risk of bias assessment is a critical step in systematic and scoping reviews, as it ensures methodological rigor and enhances the reliability of findings, reducing the likelihood of distorted or inconsistent interpretations that may not reflect reality (Sheth et al., 2024). Studies that fail to meet essential criteria for methodological validity – such as blinding of evaluators – may overestimate effects by up to 29% (Salazar et al., 2025). Therefore, although the sample predominantly comprises studies with satisfactory methodological quality regarding bias risk, the identified limitations underscore the need for improvement, particularly in blinding procedures and characterization of outcome assessors, to strengthen the robustness and credibility of future research findings.

### Plant extracts and species used for GIC modification

A total of 23 plant extracts were used across the selected studies, with a predominance of alcoholic extracts (*n* = 9; 39.1%) (Singer & Bourauel, 2021; Singer et al., 2020a, 2020b) and aqueous extracts (*n* = 6; 26.1%) (Ashour et al., 2022; Devi et al., 2022; Paulraj & Nagar, 2020). Some studies employed more than one species to enhance the formulation, resulting in 12 different plant species in total. The most frequently used was *Salvadora persica* (*n* = 6; 26.1%), reported in the studies by Ashour et al. (2022); El-Tatari et al. (2011); Siddiqui et al. (2024); Singer et al. (2020a); Singer et al. (2020b); and Singer and Bourauel (2021).

**Figure 4:**
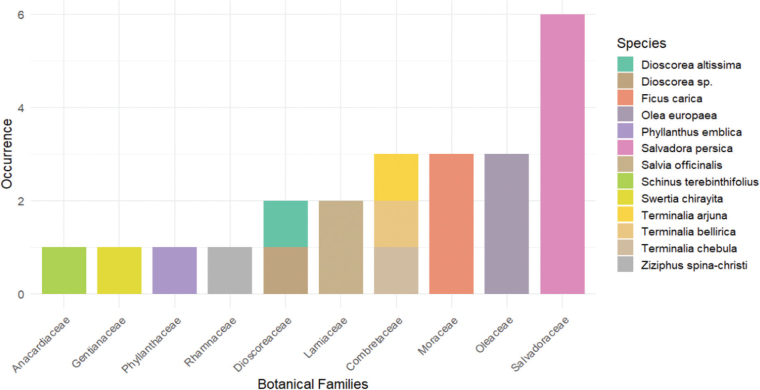
Plant species used to enhance the properties of Glass Ionomer Cements (GICs).

The extracts were derived from 10 distinct botanical families. Salvadoraceae was the most frequent (*n* = 6; 26.1%) (Ashour et al., 2022; El-Tatari et al., 2011; Siddiqui et al., 2024; Singer & Bourauel, 2021; Singer et al., 2020a, 2020b), followed by Moraceae and Oleaceae, each with three records (*n* = 3; 13.0%) (Singer et al., 2020a, 2020b) (Figure 4).

*S. persica*, commonly known as miswak or the toothbrush tree, holds significant ethnobotanical value in oral healthcare (Aljarbou et al., 2022). Its roots are traditionally used as ‘chewing sticks’ and for the fabrication of bristle-like fibres, and it is found in both African and South American regions (Aljarbou et al., 2022). It has been extensively studied in dentistry due to its antimicrobial, antioxidant, and biofilm-reducing properties (Aljarbou et al., 2022).

Studies by Ashour et al. (2020), El-Tatari et al. (2011), Siddiqui et al. (2024), and Singer et al. (2020b) demonstrated that *S. persica* extracts enhance the antibacterial properties of GICs without compromising their mechanical properties when used at concentrations below 4%. The antibacterial efficacy of *S. persica*-modified GICs was comparable to that of GICs supplemented with CHX. However, the combined extract with other species (*Olea europaea* and *Ficus carica*) caused discoloration of the cement, compromising its aesthetic properties (Singer et al., 2020a, 2020b). This effect was not reported in studies using *S. persica* alone (Ashour et al., 2022; El-Tatari et al., 2011; Siddiqui et al., 2024; Singer & Bourauel, 2021; Singer et al., 2020a) and therefore warrants further investigation.

Beyond its ethnobotanical significance, *S. persica*, like other medicinal plants, represents an economically viable and sustainable alternative. The natural raw materials used in the modification of GICs are widely available in biodiversity-rich developing countries, offering opportunities to support local economies through the trade of native plant species (Aljarbou et al., 2022).

Beyond the most frequently cited species, a diverse range of other plant extracts has been incorporated into GICs, highlighting the rich botanical diversity explored in this research. Noteworthy examples include the Combretaceae family, with the *Terminalia* genus (*n* = 3; 13.0%) (Devi et al., 2022; Paulraj & Nagar, 2020), and *Ficus carica* (Moraceae; *n* = 3; 13.0%) (Singer & Bourauel, 2021; Singer et al., 2020a, 2020b), both employed as aqueous and alcoholic extracts in their formulations. *Olea europaea* (Oleaceae; *n* = 3; 13.0%) (Singer & Bourauel, 2021; Singer et al., 2020a, 2020b), *Dioscorea altissima* (*n* = 2; 8.7%) (Chiode et al., 2022; Saraceni et al., 2019), and *Salvia officinalis* (Lamiaceae; *n* = 2; 8.7%) (Forouzanmehr et al., 2020; Shahriari et al., 2019) have also shown promise in experimental studies.

Furthermore, several other species, albeit less frequently reported, were investigated. These include *Schinus terebinthifolius* (*n* = 1; 4.3%) (Pinto et al., 2020), *Swertia chirayita* (*n* = 1; 4.3%) (Devi et al., 2022), *Phyllanthus emblica* (*n* = 1; 4.3%) (Paulraj & Nagar, 2020), and *Ziziphus spina-christi* (*n* = 1; 4.3%) (Mathew & Sghaireen, 2020). The inclusion of these plants, often sourced from distinct biomes and botanical families, not only broadens the repertoire of options for enhancing GICs but also underscores the considerable, and as yet underexploited, potential of plant biodiversity for innovation in dental materials

### Plant extracts and glass ionomer cements

**Figure 5:**
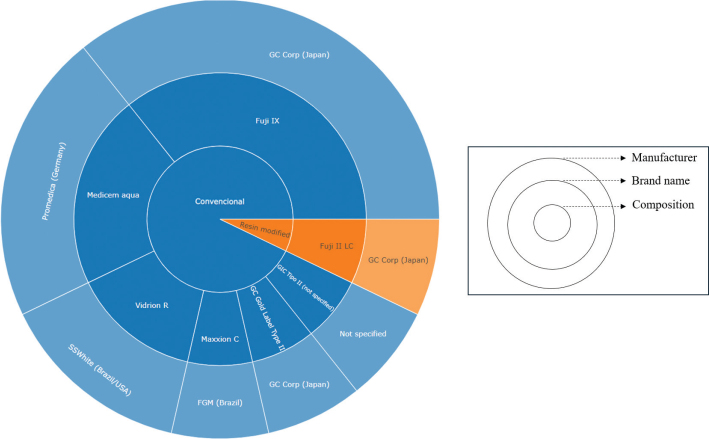
Brands and manufacturers of Glass Ionomer Cements modified with plant extracts..

The included studies employed a variety of GIC types and commercial brands (Figure 5), with a predominance of conventional GICs (*n* = 13; 92.9%) (Ashour et al., 2022; Chiode et al., 2022; Devi et al., 2022; El-Tatari et al., 2011; Mathew & Sghaireen, 2020; Paulraj & Nagar, 2020; Pinto et al., 2020; Saraceni et al., 2019; Shahriari et al., 2019; Siddiqui et al., 2024; Singer & Bourauel, 2021; Singer et al., 2020a, 2020b). Among them, the most frequently used brands were Fuji IX (GC Corporation, Japan), used in five studies (35.7%) (Ashour, 2022; Devi, 2022; El-Tatari, 2011; Paulraj, 2020; Shahriari, 2019); Promedica (Germany), used in three studies (21.4%) (Singer & Bourauel, 2021; Singer et al., 2020a, 2020b); and Vidrion R (SSWhite, Brazil), used in two studies (14.3%) (Chiode et al., 2022; Saraceni et al., 2019). Only one study, by Forouzanmehr et al. (2020), employed a resin-modified GIC.

The plant-extract-modified GICs identified in the literature were evaluated for their antimicrobial activity, as well as chemical, physical, and mechanical properties.

A notable source of potential bias is the lack of methodological details regarding the preparation, proportions, and methods of extract incorporation into the GICs. This omission diminishes the accuracy of the analyses and may lead to erroneous results and misleading interpretations (Salazar et al., 2025). Scientific reproducibility and integrity are increasingly emphasized in contemporary research (Diaba-Nuhoho & Amponsah-Offeh, 2021). The absence of key methodological steps compromises study reproducibility and limits the ability to replicate or expand upon the initial findings (Diaba-Nuhoho & Amponsah-Offeh, 2021).

According to the methodologies described, 57.14% of the studies (*n* = 8) (Ashour et al., 2022; Devi et al., 2022; Forouzanmehr et al., 2020; Paulraj & Nagar, 2020; Siddiqui et al., 2024; Singer & Bourauel, 2021; Singer et al., 2020a, 2020b) did not report how the extracts were incorporated into the GIC. The remaining 42.6% (*n* = 6) described manual homogenization as the technique used for incorporation (Chiode et al., 2022; El-Tatari et al., 2011; Mathew & Sghaireen, 2020; Pinto et al., 2020; Saraceni et al., 2019; Shahriari et al., 2019).

Extracts were incorporated at low concentrations – up to 5% – in 51.72% of the cases (*n* = 15) (Ashour et al., 2022; Chiode et al., 2022; El-Tatari et al., 2011; Forouzanmehr; Barekatain & Shahtalebi, 2020; Mathew & Sghaireen, 2020; Shahriari et al., 2019; Siddiqui et al., 2024). In terms of the phase of addition, 51.72% (*n* = 15) of the studies incorporated the extracts into the powder (w/w) (Ashour et al., 2022; Chiode et al., 2022; Forouzanmehr et al., 2020; Shahriari et al., 2019), 34.5% (*n* = 10) into the liquid (v/v) (El-Tatari et al., 2011; Siddiqui et al., 2024; Singer et al., 2020a), and 13.8% (*n* = 4) used a mixed method (w:v:w) (Devi et al., 2022; Paulraj & Nagar, 2020).

### Antimicrobial activity of GICs modified with plant extracts

GICs are characterized by inherent surface porosity and limited antimicrobial activity, conditions that favour bacterial colonization. This highlights the importance of investigating how the antibacterial properties of plant extracts might help overcome such limitations (Wulandari et al., 2022). In this regard, 11 out of the 14 reviewed studies analyzed the effects of plant extracts on microbial activity (Ashour et al., 2022; Chiode et al., 2022; Devi et al., 2022; El-Tatari et al., 2011; Mathew & Sghaireen, 2020; Paulraj & Nagar, 2020; Pinto et al., 2020; Saraceni et al., 2019; Shahriari et al., 2019; Siddiqui et al., 2024; Singer et al., 2020b).

**Figure 6:**
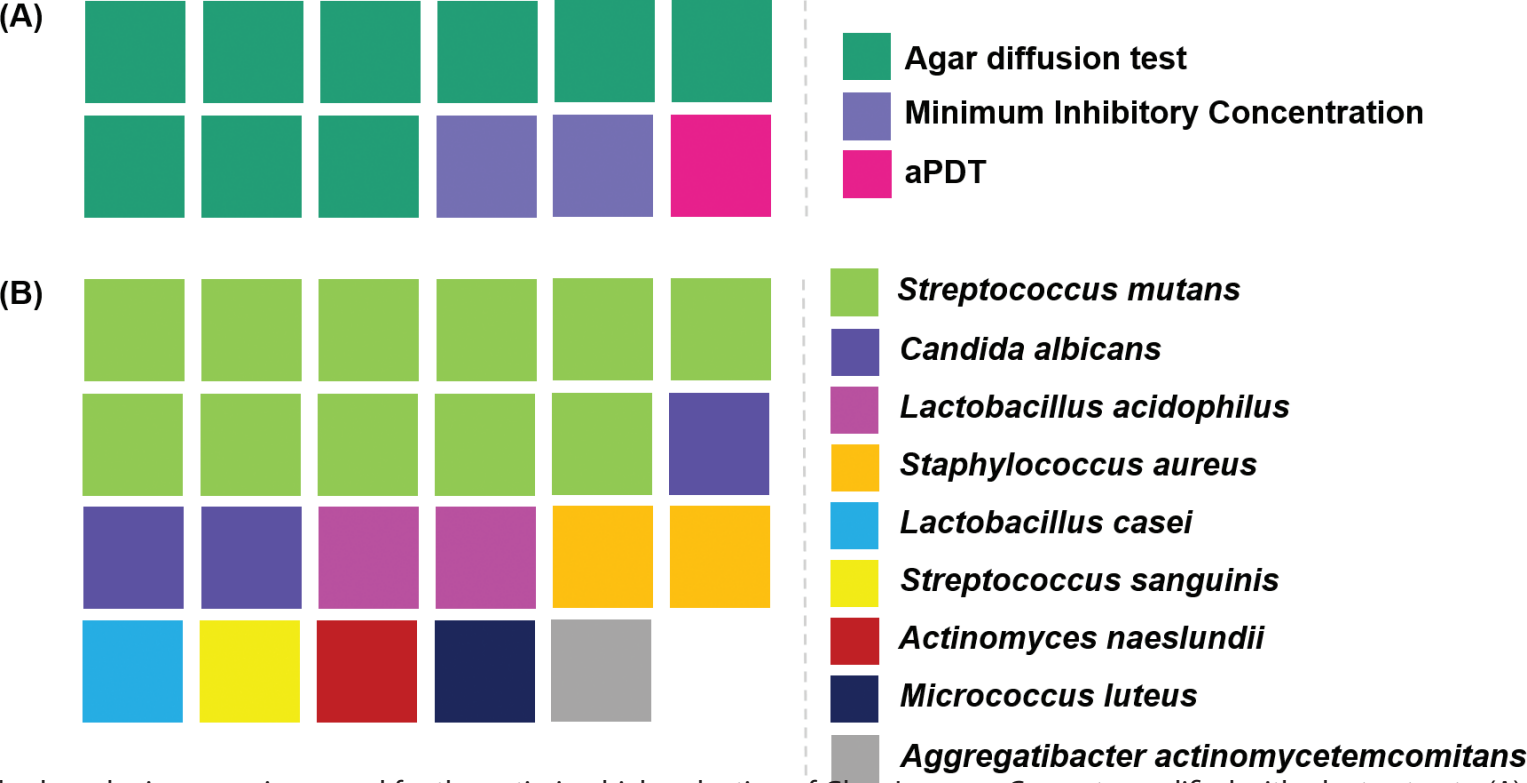
Methods and microorganisms used for the antimicrobial evaluation of Glass Ionomer Cements modified with plant extracts. (A) Methods used; (B) Microorganisms evaluated.

The studies evaluating antimicrobial activity employed one or more of the following methods (Figure 6): agar diffusion test (*n* = 9; 75%) (Ashour et al., 2022; Devi et al., 2022; El-Tatari et al., 2011; Mathew & Sghaireen, 2020; Paulraj & Nagar, 2020; Pinto et al., 2020; Shahriari et al., 2019; Siddiqui et al., 2024; Singer et al., 2020b), MIC (*n* = 2; 16.7%) (Devi et al., 2022; Saraceni et al., 2019), and aPDT (*n* = 1; 8.3%) (Chiode et al., 2022). The most commonly tested microorganisms were *Streptococcus mutans*, evaluated in all 11 studies (100.0%), and *Candida albicans*, assessed in three studies (27.3%) (Ashour et al., 2022; El-Tatari et al., 2011; Pinto et al., 2020).

Antimicrobial assays revealed that the modification of GICs with plant extracts generally led to a significant increase in antimicrobial activity against oral microorganisms. Siddiqui et al. (2024) reported the largest inhibition zones, reaching 26 mm at 3% extract concentration, surpassing even 5% CHX-modified GIC (21 mm). Paulraj and Nagar (2020) obtained promising results using Triphala, with inhibition zones of 11–13 mm, outperforming conventional GIC (5–6 mm). El-Tatari et al. (2011) observed up to a twofold increase in microbial inhibition with increasing extract concentration; *C. albicans* was inhibited only at 4%. Devi et al. (2022) emphasized the superior efficacy of *Swertia chirayita* extract over *T. arjuna* (*p* < 0.05) in inhibiting *Lactobacillus acidophilus*. Shahriari et al. (2019) reported significant inhibition of *S. mutans* at all tested concentrations (0.5–1.25%) and of *L. casei* from 0.75% upwards.

The unanimous decision among researchers to investigate GICs modified with plant extracts against *S. mutans* is well-founded, given its central role in the cariogenic process (Cai & Kim, 2023). However, a recurrent methodological limitation in several studies was the absence of dual-species and cross-kingdom analyses, which is noteworthy considering the polymicrobial nature of the oral cavity. Multispecies biofilms are widely acknowledged in the pathogenesis of dental caries (Conti et al., 2023). Overlooking the complexity of the oral microbiota reduces the validity of extrapolations from these findings, highlighting the necessity of testing GICs under conditions more representative of the oral environment (Heersema & Smyth, 2019).

Among the 11 studies, only El-Tatari et al. (2011), Ashour et al. (2022), and Saraceni et al. (2019) included fungal assessments. The methanolic extract from *S persica* roots, when incorporated into GIC, led to a reduction in bacterial inhibition zones across all tested concentrations compared to unmodified GIC. Nevertheless, a positive antifungal effect against *C. albicans* was observed solely at the 4% concentration (El-Tatari et al., 2011). This finding underscores the relevance of cross-kingdom studies. The bioactive metabolites present in *S. persica* are responsible for its antimicrobial properties against oral pathogens and reduced microbial adhesion potential, making it a promising, less aggressive alternative to conventional chemical agents (Aljarbou et al., 2022).

All plants contain a wide variety of phenolic compounds, which may exert anti-adherent effects through interactions with microbial proteins, as well as broad-spectrum antimicrobial activity (Flemming et al., 2021). In addition, other classes of specialized plant metabolites, such as terpenes and saponins, also exhibit significant antimicrobial potential (Linhares et al., 2022). Therefore, studies incorporating plant extracts into GICs should include a phytochemical characterization of the material to identify the bioactive compounds present and to assess their potential release over time.

### Cytotoxic characterization of GICs modified with plant extracts

Among the studies investigating the incorporation of plant extracts into GICs, only the study by Pinto et al. (2020) included a cytotoxicity assessment of the modified material. This lends uniqueness and scientific robustness to the research, as biocompatibility is a fundamental requirement for any biomaterial intended for clinical use and remains one of the hallmark properties of GICs (Ersahan et al., 2020; Makanjuola & Deb, 2023; Nicholson et al., 2023; Song et al., 2025). The study examined *Schinus terebinthifolius* and evaluated the effects of its ethanolic extract when incorporated into the cement. While the pure extract exhibited low to moderate cytotoxicity, with cell viability below 50%, the GIC containing the extract did not demonstrate any relevant cytotoxic effect, maintaining cell viability above 70%. Although the extract’s toxicity was attenuated by its incorporation into the cement matrix, the resulting material still showed reduced cell viability when compared to the unmodified GIC.

The absence of cytotoxic characterization of these cements in the literature represents a critical gap in current research. Given that plant extracts contain a variety of bioactive compounds, some of which may not be biocompatible with oral tissues, such assessments are of paramount importance (Song et al., 2025). This concern becomes even more significant considering that GICs often remain in close proximity to, or in direct contact with, sensitive structures such as the dental pulp and dentin for extended periods (Makanjuola & Deb, 2023). Therefore, the evaluation of cytotoxic effects in GICs modified with plant-derived substances is imperative.

## Physicochemical characterization of GICs modified with plant extracts

The majority of studies – nine out of 14 (64.3%) (Devi et al., 2022; El-Tatari et al., 2011; Forouzanmehr et al., 2020; Mathew & Sghaireen, 2020; Paulraj & Nagar, 2020; Shahriari et al., 2019; Siddiqui et al., 2024; Singer & Bourauel, 2021; Singer et al., 2020b) – did not describe any method for chemical characterization. This omission significantly limits the interpretability of the findings, as it precludes direct correlations between the observed effects and the chemical constituents present in the materials (Pang et al., 2021).

Among the five studies that conducted chemical characterization (Ashour et al., 2022; Chiode et al., 2022; Pinto et al., 2020; Saraceni et al., 2019; Singer et al., 2020), the primary objective in 85.7% of them was molecular identification (Ashour et al., 2022; Chiode et al., 2022; Pinto et al., 2020; Singer et al., 2020). These studies employed techniques such as Gas Chromatography–Mass Spectrometry (GC-MS) and Ultra-Performance Liquid Chromatography coupled with Quadrupole Time-of-Flight Tandem Mass Spectrometry (UPLC-QTOF-MS/MS). Chromatographic analyses consistently identified the presence of phenolic compounds – primarily tannins and flavonoids – reflecting their widespread bioavailability and their well-documented antibacterial, antioxidant, and anti-inflammatory properties (Elshafie et al., 2023). In addition, spectrophotometric methods such as UV–Vis were used to evaluate the absorbance of compounds within the GICs, and Fourier Transform Infrared Spectroscopy (FTIR) was employed to examine the functional groups of the organic compounds present in the extracts, identifying components such as phenolic groups and flavonoids in the ethanolic extract of *S. terebinthifolius* Raddi fruits.

One of the key advantages of GICs is their ability to release ions such as fluoride and calcium, which promote dental remineralization. Therefore, it is essential to assess whether the incorporation of plant extracts interferes with this property (Nicholson et al., 2023). Plant-derived additives are emerging as alternatives to conventional agents aimed at enhancing the antimicrobial properties of GICs, especially considering that CHX has been shown to reduce this beneficial ion release (Hassan et al., 2022). Nonetheless, only Saraceni et al. (2020) investigated fluoride release in a GIC modified with *Dioscorea altissima* extract and reported no impairment in this regard. No study to date has assessed the release of calcium or other ions, which represents a significant gap in the literature given the crucial role of these ions in the overall performance of GICs.

**Figure 7:**
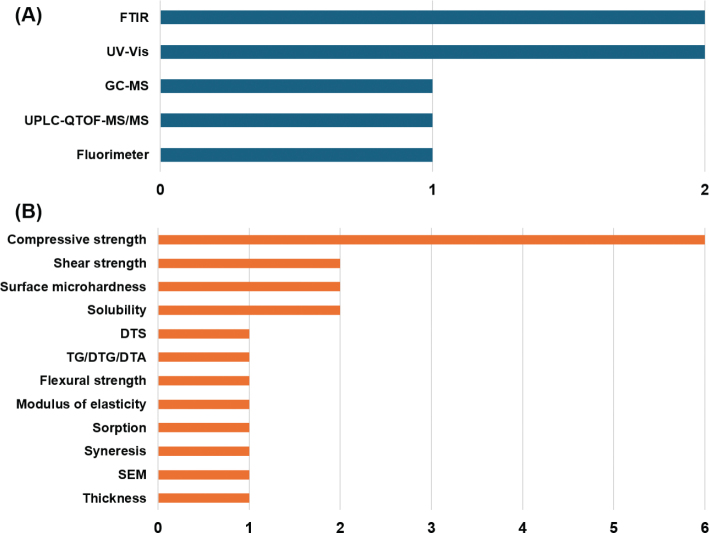
Physicochemical assessment methods of glass ionomer cements modified with plant extracts. (A): chemical characterization; (B): physical characterization..

Physical characterization of GICs was reported in 10 of the 14 selected studies (71.4%) (Figure 7), including the works by Ashour et al. (2022), Devi et al. (2022), El-Tatari et al. (2011), Forouzanmehr, Barekatain and Shahtalebi (2020), Pinto et al. (2020), Saraceni et al. (2019), Siddiqui et al. (2024), Singer et al. (2020), Singer et al. (2020b), and Singer and Bourauel (2021). It is important to highlight that GIC additives must preserve essential physical properties including strength, elasticity, and surface roughness to ensure restoration durability (Gomes et al., 2024). Most studies prioritized the evaluation of mechanical properties (65.0%) (Ashour et al., 2022; Devi et al., 2022; El-Tatari et al., 2011; Forouzanmehr et al., 2020; Pinto et al., 2020; Saraceni et al., 2019; Siddiqui et al., 2024; Singer & Bourauel, 2021; Singer et al., 2020, 2020b). CS was tested in six studies following ISO 9917-1 standards, which stipulate minimum values of ≥100 MPa for restorative use and ≥ 50 MPa for base or luting applications (Técnicas, 2019). A mixed extract of *S. persica*, *O. europaea*, and *F. carica* significantly enhanced CS to 86.2 MPa at a 2:1 extract-to-water ratio, outperforming both unmodified GIC (63.8 MPa) and GIC containing 0.5% CHX (63 MPa) (Singer et al., 2020b). Conversely, ethanolic extract of *S. terebinthifolius* Raddi reduced CS by 9%, yielding 176.60 ± 6.46 MPa compared to the control GIC (161.17 ± 5.01 MPa) (Pinto et al., 2020). Other tested extracts did not enhance CS but maintained mechanical integrity without compromising material performance. The improvement in CS was attributed to the presence of silica in the *S. persica* extract, which may chemically bond to the matrix and reinforce the GIC structure (Singer et al., 2020b).

Water stability is a critical factor in the clinical success of GIC-based restorations due to the material’s hydrophilic nature, which makes it susceptible to degradation processes such as imbibition, syneresis, and solubility (Song et al., 2025). These aspects were addressed in only 15.0% of studies reporting physical characterization. Compared to conventional GICs, extract-modified materials demonstrated improved or equivalent performance. Notably, the 2:1 extract-to-water ratio of the *S. persica, O. europaea*, and *F. carica* formulation showed reduced solubility relative to lower concentrations, supporting the hypothesis that higher extract concentration enhances chemical stability (Singer et al., 2020).

Despite these findings, the literature still lacks comprehensive evaluations of several mechanical properties. Few studies explore the full physical behaviour of GICs modified with plant extracts in comparison to CHX, the most studied antimicrobial additive, highlighting the need for further research to understand potential advantages (Gomes et al., 2024). Though less frequently reported, additional mechanical tests provided important insights. Shear bond strength was assessed by Forouzanmehr et al. (2020) and Singer and Bourauel (2021), with the latter reporting superior values in extract-modified groups compared to CHX (1.7 MPa), particularly for the 1:2 ratio group (5.1 MPa). Regarding microhardness, Saraceni et al. (2019) observed a significant increase with 2% *Dioscorea altissima* (*p* = 0.0001), while Siddiqui et al. (2024) reported higher values in GIC with 3% *S. persica* (41.10 ± 2.08 VHN) compared to conventional GIC (36.60 ± 2.59 VHN). Concerning elastic modulus, Saraceni et al. (2019) found no significant change (*p* > 0.05) upon adding 2% *D. altissima*, maintaining appropriate rigidity. Finally, flexural strength evaluated by Singer et al. (2020) showed that the lowest extract concentration (1:2) resulted in the weakest values (< 12 MPa), comparable to the control group and indicating no structural reinforcement.

Another critical limitation identified was the lack of assessment regarding loading efficiency and the substantivity of extract incorporation. No study quantified the actual retention of active compounds within the cured GIC matrix, nor did they monitor in vitro or in vivo release kinetics. This gap severely impacts the clinical translatability of current findings, as early leaching or degradation of extracts may compromise long-term efficacy (Salazar et al., 2025). Future research should prioritize these analyses, particularly under conditions that simulate the oral environment (variable pH, mechanical abrasion, salivary flow).

Notably, there is a marked lack of in vivo studies on GICs modified with plant extracts, in contrast to other additives. This includes all levels of clinical research, from laboratory models simulating the oral environment to *in situ* studies and formal clinical trials. Although *in vitro* findings are promising, substantial future research is required to fully elucidate the biocompatibility, bioactivity, and antimicrobial efficacy of these materials under clinically relevant conditions.

One frequently noted drawback was the optical alteration of GIC due to extract incorporation, as reported by Saraceni et al. (2019), Singer et al. (2020a), and Singer et al. (2020b). The dark pigmentation of plant extracts led to noticeable discoloration of the cement, potentially reducing clinical acceptance. This colour change is attributed to secondary plant metabolites – such as terpenoids and phenolics – present in the extracts, which not only impart pigmentation but also serve protective biological roles (Elshafie et al., 2023).

This systematic review was rigorously conducted to consolidate the available evidence on GIC modification using plant extracts, highlighting both the most promising findings and current methodological limitations. The growing body of literature reflects increasing interest in this field. Plant extracts – particularly *S. persica* – appear promising for enhancing GIC properties, notably antimicrobial activity, without compromising mechanical integrity. Compared to CHX, the most studied GIC additive, certain concentrations of plant extracts present advantages such as reduced risk of microbial resistance, preserved biocompatibility, and maintained physical performance, based on experimental evidence. In this context, the results discussed herein offer an overview of the current state of research on GICs and plant-based modifications, paving the way for more robust, evidence-based future investigations.

## Conclusions

The in vitro studies demonstrated that the incorporation of plant extracts into GICs enhances primarily their antibacterial activity without compromising their chemical, physical, or mechanical properties – particularly fluoride release. However, the current literature displays considerable methodological heterogeneity, including variation in extract concentrations, plant species used, and chemical-mechanical testing protocols. This diversity, combined with insufficient methodological detail, limits the reproducibility of findings.

Moreover, critical data regarding the toxicity and biocompatibility of GICs modified with plant extracts in the oral environment remain largely unexplored, thereby constraining the clinical applicability of existing findings.

Future research should prioritize standardization of methodologies and testing protocols, as well as long-term evaluations, in order to provide clinically translatable evidence for the integration of plant extracts into dental restorative materials.

## Data Availability

Data sharing is not applicable to this article as no new data were created or analyzed in this study.
